# Comparative and phylogenetic analysis of the mitochondrial genomes in basal hymenopterans

**DOI:** 10.1038/srep20972

**Published:** 2016-02-16

**Authors:** Sheng-Nan Song, Pu Tang, Shu-Jun Wei, Xue-Xin Chen

**Affiliations:** 1 State Key Laboratory of Rice Biology and Ministry of Agriculture Key Laboratory of Agricultural Entomology, Institute of Insect Sciences, Zhejiang University, Hangzhou 310058, China; 2 Institute of Plant and Environmental Protection, Beijing Academy of Agriculture and Forestry Sciences, Beijing 100097, China

## Abstract

The Symphyta is traditionally accepted as a paraphyletic group located in a basal position of the order Hymenoptera. Herein, we conducted a comparative analysis of the mitochondrial genomes in the Symphyta by describing two newly sequenced ones, from *Trichiosoma anthracinum*, representing the first mitochondrial genome in family Cimbicidae, and *Asiemphytus rufocephalus,* from family Tenthredinidae. The sequenced lengths of these two mitochondrial genomes were 15,392 and 14,864 bp, respectively. Within the sequenced region, *trnC* and *trnY* were rearranged to the upstream of *trnI*-*nad2* in *T. anthracinum*, while in *A. rufocephalus* all sequenced genes were arranged in the putative insect ancestral gene arrangement. Rearrangement of the tRNA genes is common in the Symphyta. The rearranged genes are mainly from *trnL1* and two tRNA clusters of *trnI*-*trnQ-trnM* and *trnW-trnC-trnY*. The mitochondrial genomes of Symphyta show a biased usage of A and T rather than G and C. Protein-coding genes in Symphyta species show a lower evolutionary rate than those of Apocrita. The Ka/Ks ratios were all less than 1, indicating purifying selection of Symphyta species. Phylogenetic analyses supported the paraphyly and basal position of Symphyta in Hymenoptera. The well-supported phylogenetic relationship in the study is Tenthredinoidea + (Cephoidea + (Orussoidea + Apocrita)).

A typical animal mitochondrial genome is circular with a relatively conserved gene content. It is approximately 16 kb and encodes 37 genes, including 13 protein-coding genes (PCGs), two ribosomal RNA (rRNA) genes, 22 transfer RNA (tRNA) genes, and an A+T-rich region^1^,^2^. Mitochondrial genomes are characterized by several features, such as conserved gene content and organization, a small genome size, a lack of extensive recombination, maternal inheritance, and an accelerated rate of nucleotide substitution^3^,^4^,^5^. Consequently, this small molecule has been widely used for phylogenetic analyses of many groups^2^,^3^,^6^,^7^,^8^.

The Hymenoptera (sawflies, wasps, bees and ants) is one of the largest insect orders based on the number of described species^9^,^10^, which today exceeds 115,000. Because of their extensive distribution, wide diversity, and high economic importance^9^,^10^, there has been increased interest in the evolutionary history of the order Hymenoptera^10^. However, the phylogenetic relationships remain largely unclear^11^. The monophyly of the Hymenoptera is well supported by studies based on morphological data^12^, molecular data^13^,^14^, and both in combination^15^,^16^. The Hymenoptera are traditionally divided into two suborders: Symphyta and Apocrita. The Symphyta is generally considered a paraphyletic lineage, and is often identified by a “broad-waist”. The other suborder, Apocrita, is presumably monophyletic, and they all share the feature of a constricted “wasp-waist”^9^,^10^,^16^.

The Symphyta has long been recognized at a basal position of the Hymenoptera^9^,^10^,^13^,^14^,^16^,^17^,^18^,^19^. This suborder consists of seven extant superfamilies: Xyeloidea, Pamphilioidea, Tenthredinoidea, Cephoidea, Siricoidea, Xiphydrioidea, and Orussoidea^10^. There have been many phylogenetic studies of Symphyta in the past several years, based on morphology^18^,^19^, DNA sequences^9^, or a combination of both approaches^20^,^21^. The monophyly of Tenthredinoidea and Pamphilioidea, the basal position of Xyeloidea, the main relationships between the superfamilies, and the sister relationship between Orussoidea and Apocrita have been confirmed by many analyses^9^,^19^,^20^,^21^. However, there are still many unclear questions among the Symphyta and Tenthredinoidea, such as the position of Pamphilioidea, the sister group of Tenthredinoidea, and the phylogenetic relationships among Tenthredinoidea^19^,^20^,^21^. Within the Tenthredinoidea, the monophyly of Tenthredinidae is unclear with regard to Cimbicidae and Diprionidae^16^,^21^.

Mitochondrial genomes have been widely used for phylogenetic analysis of the Hymenoptera^13^,^14^. Mitochondrial genomes in the Hymenoptera have been found to show many unique characteristics, such as an extremely high rate of evolution^22^,^23^,^24^,^25^, frequent gene rearrangement^26^,^27^, and a strong base composition bias^28^,^29^,^30^. However, most of the studies have mainly been related to species from the Apocrita. Until now, complete or nearly complete mitochondrial genomes have only been reported for eight species in the Symphyta. Mitochondrial genomes in the Symphyta have been generally considered to be ordinary^31^, as in most other insect orders^3^,^32^, compared with the ones from Apocrita. Nevertheless, gene rearrangement has frequently been found in the Symphyta^33^,^34^,^35^,^36^.

Here, we compared the features of the mitochondrial genomes within the Symphyta by adding two newly sequenced mitochondrial genomes of Tenthredinoidea. The first was *Trichiosoma anthracinum*, which belongs to Cimbicidae, as the first representative of Cimbicidae, and the other was *Asiemphytus rufocephalus,* belonging to the Allantinae subfamily of the Tenthredinidae. Finally, we investigated the phylogenetic relationships within Symphyta.

## Results and Discussion

### General features of the two newly sequenced mitochondrial genomes

We sequenced two nearly complete mitochondrial genomes from *T. anthracinum* (GenBank accession KT921411) and *A. rufocephalus* (GenBank accession KR703582). The sequenced region of the *T. anthracinum* mitochondrial genome was 15,392 bp long, with 13 protein-coding, two rRNA and 20 tRNA (except for *trnQ* and *trnM*) genes, and a partial A+T-rich region. There were 619 bp intergenic nucleotides in total, in 18 locations, and the length of the intergenic spacers was 1–414 bp. The longest intergenic spacer (414 bp) was located at the start of the mitochondrial genome before *trnY*. Four pairs of genes overlapped each other, with a length ranging from 2 to 7 bp. Fourteen pairs of genes were directly adjacent.

The sequenced mitochondrial genome of *A. rufocephalus* was 14,864 bp long, with 13 protein-coding, two rRNA and 19 tRNA genes, and a partial A+T-rich region. Part of the A+T-rich region and three tRNA genes (*trnI*, *trnQ* and *trnM)* failed to be sequenced. There were 150 bp intergenic nucleotides in total, in 16 locations, and the length of the intergenic spacers was 1–27 bp. The longest intergenic spacer (27 bp) was located between *cox3* and *trnG*. Five pairs of genes overlapped each other, with a length ranging from 1 to 8 bp. Thirteen pairs of genes were directly adjacent.

### Gene rearrangement in the Symphyta

**Figure 1:**
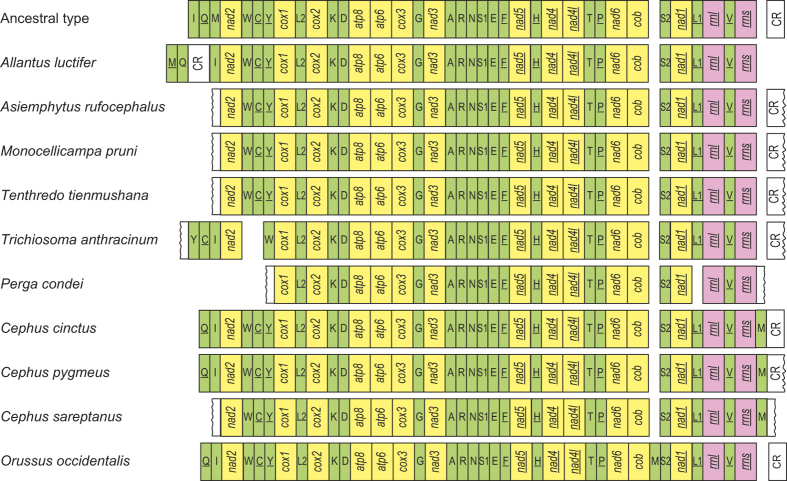
Mitochondrial genome organization of Symphyta referenced with the ancestral insect mitochondrial genomes. Genes are transcribed from left to right except for those indicated by a line. PCGs, rRNA, and A+T-rich region genes are marked in yellow, pink, and white, respectively, while tRNA genes are marked in green and designated by the single-letter amino acid code.

Compared with the putative ancestral gene arrangement of insects, there have been at least two rearrangement events in the mitochondrial genome of *T. anthracinum*, corresponding to the remote inversion of *tnnY* and the translocation of *trnC* from a location between *trnW* and *cox1* to upstream of *trnI*. There was no gene rearrangement in the sequenced region of *A. rufocephalus* (Fig. 1). However, we could not exclude the possibility of gene rearrangement in the unsequenced tRNA cluster *trnI*-*trnQ*-*trnM*.

In the sequenced mitochondrial genomes of 10 Symphyta species (first 10 species in Table 1), gene rearrangement was found in seven species. No rearranged genes were found in the sequenced region of *A. rufocephalus*, *Monocellicampa pruni,* and *Tenthredo tienmushana*, in which the tRNA cluster *trnI-trnQ-trnM* is not detected. There was no PCG rearrangement in the Symphyta mitochondrial genomes. The rearranged tRNA genes were mainly from the tRNA clusters of *trnI*-*trnQ*-*trnM* and *trnW*-*trnC*-*trnY*, and *trnL1* in *Perga condei*. Compared with the mitochondrial genomes of Apocrita, gene rearrangement is relatively conserved in the suborder Symphyta^3^,^14^; however, it is frequent compared with most other insect orders^37^,^38^,^39^,^40^.

### Base composition

**Figure 2:**
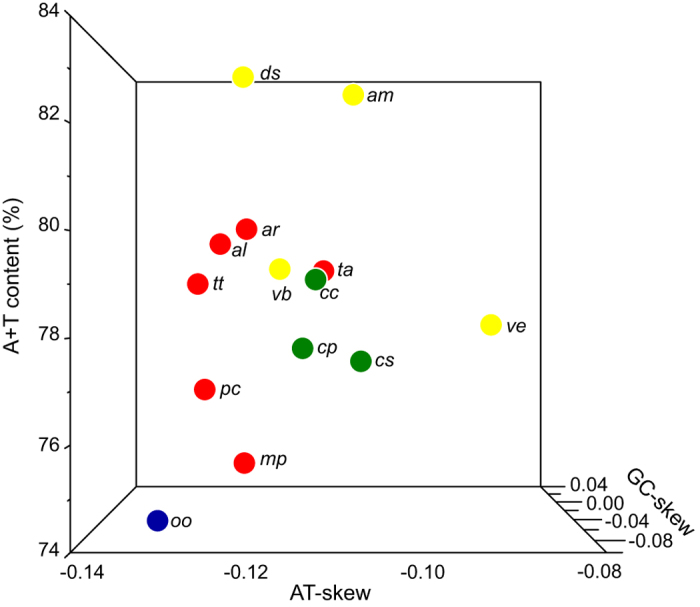
Three-dimensional scatter-plot of the AT- and GC-skew and A+T% of the mitochondrial genomes of Symphyta. Species of Tenthredinoidea are represented by red dots, Cephoidea by green dots, Orussoidea by blue dots, and Apocrita by yellow dots. *al*: *Allantus luctifer*; *ar*: *Asiemphytus rufocephalus*; *mp*: *Monocellicampa pruni*; *tt*: *Tenthredo tienmushana*; *ta*: *Trichiosoma anthracinum*; *pc*: *Perga condei*; *cc*: *Cephus cinctus*; *cp*: *Cephus pygmeus*; *cs*: *Cephus sareptanus*; *oo*: *Orussus occidentalis*; *am*: *Apis mellifera*; *vb*: *Vespa bicolor*; *ds*: *Diadegma semiclausum*; *ve*: *Vanhornia eucnemidarum*.

Three parameters, AT-skew, GC-skew, and A+T content, are usually used in the investigation of the nucleotide-compositional behavior of mitochondrial genomes^29^,^41^. The mitochondrial genomes of *T. anthracinum* and *A. rufocephalus* both showed a very strong bias in nucleotide composition (A+T% > G+C%), which is typical in insect mitochondrial genomes. The A+T content of the PCGs of all 10 Symphyta species was between 74.20% (*Orussus occidentalis*) and 80.30% (*A. rufocephalus*), while the A+T content of *T. anthracinum* and *A. rufocephalus* was 79.30% and 80.30%, respectively (Table 2). In the PCG sequences of Symphyta, all of the AT-skews were negative while most GC-skews were negative (except in *A. rufocephalus*, *P. condei* and *Cephus pygmeus*), indicating that the PCGs contained a higher percentage of T and C than A and G nucleotides (Table 3), as reported in most other insects^29^. The results of the comparative analysis of the A+T content and the AT- and GC-skew of the PCGs within the sequenced Symphyta mitochondrial genomes are illustrated in a three-dimensional scatter-plot chart (Fig. 2). Species rom Symphyta and Apocrita were separated, except for *V. bicolor*, which was mixed with Symphyta. Within the Symphyta, species from each of the three superfamilies were clustered, except for *T. anthracinum* from Tenthredinoidea.

### Protein-coding genes and codon usage

Among the 13 PCGs in the *T. anthracinum* and *A. rufocephalus* mitochondrial genomes, nine PCGs were located on the majority strand (J-strand), while the other four PCGs were located on the minority strand (N-strand) (Fig. 1). The entire length of the PCGs of *T. anthracinum* was 11,185 bp, while that of *A. rufocephalus* was 11,257 bp. The overall A+T content of the 13 PCGs was 79.30% in the *T. anthracinum* mitochondrial genome, ranging from 72.58% (*cox1*) to 88.27% (*atp8*). In the *A. rufocephalus* mitochondrial genome, the A+T content of the 13 PCGs was 80.30%, ranging from 73.45% (*cox1*) to 91.36% (*atp8*).

In the *T. anthracinum* and *A. rufocephalus* mitochondrial genomes, all of the PCGs started with ATN codons. However, there are abnormal start codons in the Symphyta, including GTG (*Cephus pygmeus*, *atp8*), TTG (*Cephus sareptanus*, *nad1*), and AAA (*C. pygmeus* and *C. sareptanus*, *nad2*). In the *T. anthracinum* mitochondrial genome, two, four and seven PCGs started with ATA, ATG and ATT, respectively, while in the *A. rufocephalus* mitochondrial genome, four, three and six PCGs started with ATA, ATG and ATT, respectively. The stop codons are far less variable than the start codons in Symphyta mitochondrial genomes. Most stop codons of PCGs are complete or incomplete TAA, except in the PCGs *nad1* (*A. luctifer*, *C. sareptanus*), *nad4* (*C. cinctus*, *O. occidentalis*), and *nad5* (*C. cinctus*, *P. condei*, *T. tienmushana*, *O. occidentalis*), which have TAG as the stop codon. In both the *T. anthracinum* and *A. rufocephalus* mitochondrial genomes, 12 of the 13 PCGs ended with complete TAA, while *nad5* in *T. anthracinum* and *nad4* in *A. rufocephalus* stopped with the incomplete termination codon T.

**Figure 3:**
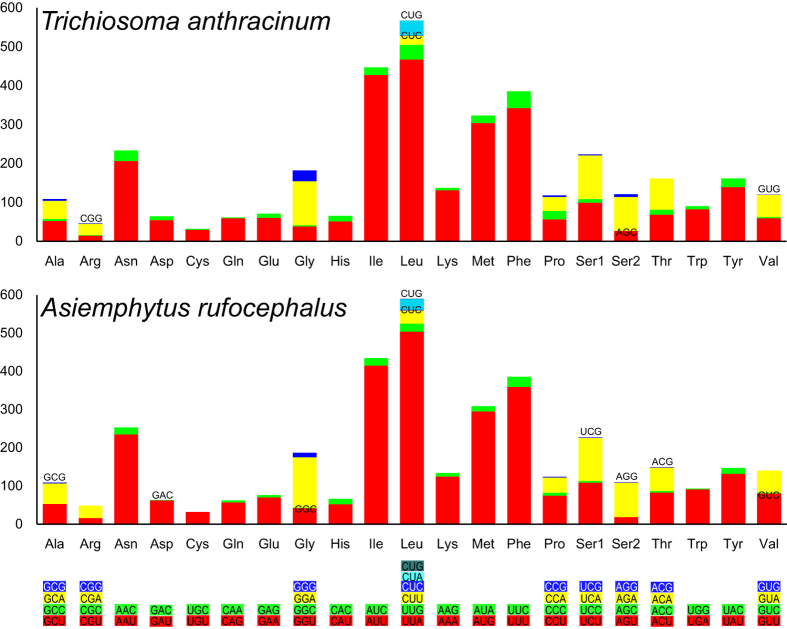
Relative synonymous codon usage (RSCU) of the mitochondrial genomes of Symphyta. The stop codon is not given.

The codon usage in the mitochondrial genomes of Symphyta also shows a significant bias towards A/T (Fig. 3). In the mitochondrial genomes of *T. anthracinum* and *A. rufocephalus*, Leu, Ile, Phe and Met were the most frequently used amino acids, while TTA (Leu), ATT (Ile), TTT (Phe) and ATA (Met) were the most frequent codons, as in other hymenopteran mitochondrial genomes^14^,^42^,^43^. All the three frequently used codons consisted of A and T, which may lead to the high A+T content in the mitochondrial genome. It is obvious that the preferred codon usage is A/T in the third position rather than G/C, as almost all of the frequently used codons ended with A/T. In the mitochondrial genome of *T. anthracinum*, the codon Thr (ACG) was missing, while Val (GTG), Ala (GCC), Cys (TGC), Arg (CGC, CGG) and Ser (AGC) were missing in the mitochondrial genome of *A. rufocephalus*. The missing codons all have a high content of G/C in the third codon position.

### Evolutionary rates of protein-coding genes

**Figure 4:**
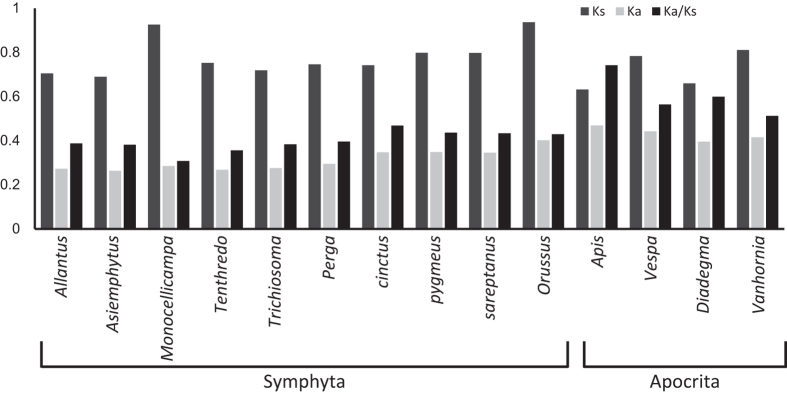
Evolutionary rates of Symphyta mitochondrial genomes. The number of nonsynonymous substitutions per nonsynonymous site (Ka), the number of synonymous substitutions per synonymous site (Ks), and the ratio of Ka/Ks for each Symphyta mitochondrial genome are given, using that of *A. appendiculatus* as a reference sequence.

The rate of nonsynonymous substitutions (Ka) and synonymous substitutions (Ks), and the ratio of Ka/Ks, were calculated for PCGs of each Symphyta mitochondrial genome using *A. appendiculatus* as a reference sequence (Fig. 4). All of the Ka and Ks values were less than 1. Simultaneously, the ratios of Ka/Ks were all less than 1, indicating the existence of purifying selection in these species. Species of Symphyta showed lower evolutionary rates than Apocrita species, as is widely accepted^34^.

### Transfer RNA and ribosomal RNA genes

The anticodons of all the tRNAs of the two newly sequenced mitochondrial genomes were identical to other Symphyta species. In the mitochondrial genome of *T. anthracinum*, the tRNA length obtained was 1359 bp, with an A+T content of 83.22%. The length of the tRNAs ranged from 64 bp (*trnT*) to 72 bp (*trnH*). In the 20 tRNA genes, 14 genes were located on the J-strand, while the others were encoded on the N-strand. In the mitochondrial genome of *A. rufocephalus*, the whole length of tRNAs obtained was 1282 bp, with a A+T content of 83.37%. The length of the tRNAs ranged from 60 bp (*trnV*) to 72 bp (*trnK*). In the 19 tRNA genes, 12 genes were located on the J-strand, while the others were encoded on the N-strand.

In the two mitochondrial genomes, all of the tRNAs could be folded into classic clover leaf structures, except for *trnV* and *trnS1*^(*AGN*)^ in *A. rufocephalus*. The dihydrouridine (DHU) arm of these two tRNAs was a large loop instead of the conserved stem-and-loop structure, which is a typical feature of metazoan mitochondrial genomes^44^. All the secondary structures of the tRNA genes could be predicted by Mitos WebServer^45^. In the tRNAs of the two mitochondrial genomes, the amino acid acceptor (AA) stem and the anticodon (AC) loop were conserved as 7 bp, except for the AC-loops of *trnL2*^(*CUN*)^ and *trnR* in *T. anthracinum* and *trnL2*^(*CUN*)^ in *A. rufocephalus*, which were 9 bp. The length of tRNA usually depends on the size of the variable loop and the D-loo^46^. The DHU arm was 3–4 bp and the AC arm was 4–5 bp, while the TΨC arm varied from 3–5 bp. The variable loops were less consistent, ranging from 4–10 bp.

In the mitochondrial genomes, some base pairs were not the classic A-U and C-G, based on the secondary structure. Five mismatched base pairs were found in the tRNAs of each of *T. anthracinum* and *A. rufocephalus*. Among the five mismatched base pairs of *T. anthracinum*, four of them were U-U pairs located in the AA stem, while the other was an A-C pair located in the TΨC stem (Table 4). However, in *A. rufocephalus*, there were three U-U pairs located in the AA stem and one U-U pair in the AC stem, and the other was an A-A pair located in the TΨC stem (Table 4).

In the mitochondrial genome of *T. anthracinum*, the *rrnL* was 1351 bp long with an A+T content of 84.46%, while the *rrnS* was 800 bp long with an A+T content of 83.50%. In the mitochondrial genome of *A. rufocephalus*, the *rrnL* was 1339 bp long with an A+T content of 85.06%, while the *rrnS* was 803 bp long with an A+T content of 83.44%. In the 10 Symphyta species, the length of *rrnS* ranged from 728 (*P. condei*) to 1014 bp (*C. sareptanus*), while the length of *rrnL* ranged from 1336 (*O. occidentalis*) to 1386 bp (*C. cinctus*).

### Intergenic spacers and overlapping regions

We identified 20 intergenic spacers in the *T. anthracinum* mitochondrial genome, and 17 intergenic spacers in the *A. rufocephalus* mitochondrial genome. In the *T. anthracinum* mitochondrial genome, the longest intergenic spacer was located at the start of the mitochondrial genome before *trnY* and the second longest region was the incomplete A+T-rich region with a length of 100 bp, while the other 18 regions ranged from 1 to 47 bp. In the mitochondrial genome of *A. rufocephalus*, the longest intergenic spacer was also the incomplete A+T-rich region, with a length of 55 bp, and the other 16 regions ranged from 1 to 27 bp. In both species, we did not sequence the full length of the A+T-rich region. The content of these two incomplete A+T-rich regions was 78% and 85.45%, respectively.

There was an “ATTATAA” motif between *nad4* and *nad4l* in the N-strand of the *T. anthracinum* mitochondrial genome, but this intergenic region did not exist in the *A. rufocephalus* mitochondrial genome, because of the direct adjacency of *nad4* and *nad4l*. There was an overlap sequence of “ATGATAA” present between *atp8* and *atp6*. This characteristic 7 bp overlap of the two PCGs located on the J-strand appears in most species of Symphyta and other insects.

### Phylogenetic relationships

**Figure 5:**
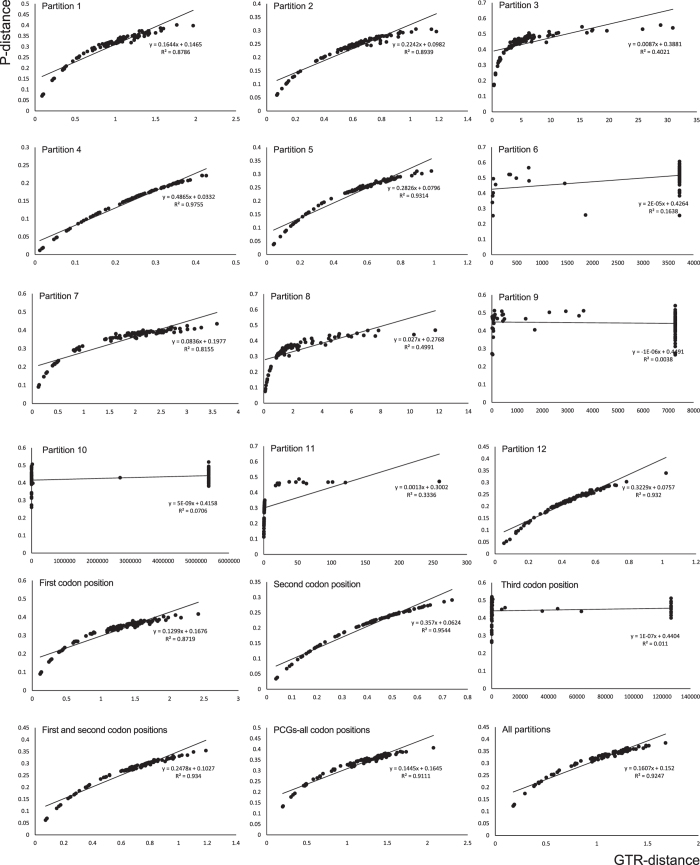
Scatter-plot graphics performed to test the saturation of the nucleotide substitution in the mitochondrial genomes in Symphyta. Pairwise distances were calculated for the 12 partitions, the first, second and third codon positions of the 13 PCGs and all the partitions’ concatenated matrix.

**Figure 6:**
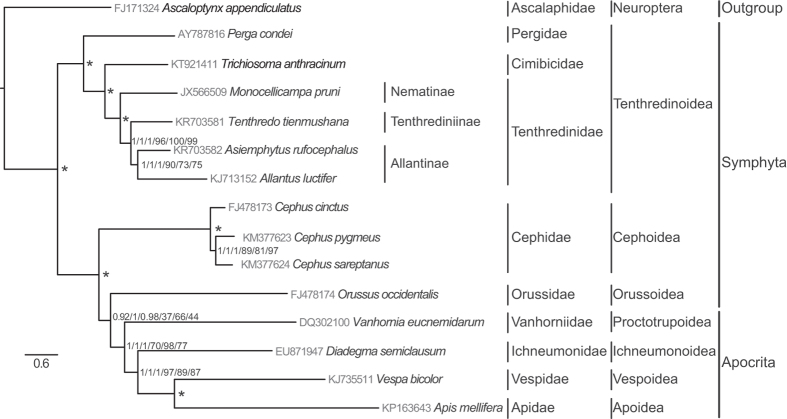
Phylogenetic relationships of the Symphyta inferred from nucleotide sequences of the mitochondrial genome using three matrices (P123, P123R and P12T), referring to the Bayesian/Maximum Likelihood methods. The numbers separated by “/” indicate the posterior probability and bootstrap values of the corresponding nodes (P123 using BI/P123R using BI/P12T using BI/P123 using ML/P123R using ML/P12T using ML). “*” indicates that the node was fully supported by all six inferences.

We reconstructed the phylogeny among the Symphyta (Fig. 5). Before reconstructing the phylogenetic tree, the saturation of different partitions generated from PartitionFinder was tested (Fig. 6). The results indicated that partitions 6, 9, 10 and 11 identified by PartitionFinder had experienced substitution saturation. From the results of the data partitioning, we found that these four partitions contained all the third positions of PCGs and two rRNA genes. We also tested the saturation of all partitions and different codon positions in the PCGs (Fig. 6). The results showed that substitution saturation occurred in the third position. Hence, we reconstructed the phylogenetic relationship using four data matrices, i.e. 11,931 sites in the P123 matrix (containing the three codon positions of PCGs), 16,548 sites in the P123R matrix (containing the three codon positions of PCGs, two rRNA genes and 20 tRNA genes), 9483 sites in the P12T matrix (excluding partitions 6, 9, 10 and 11) and 3977 sites in the AA matrix (containing the amino acid sequences).

For Bayesian and ML analyses, all matrices of nucleotide sequences (P123, P123R and P12T) generated the same topology with high posterior probabilities. However, the matrix of the amino acid sequences generated from both BI and ML analyses showed a different topology to that of the nucleotide sequences, in which Cephoidea forms a sister group with Orussoidea, and then these two groups form a sister group to Apocrita. These topologies have not been reported in previous studies^9^,^20^,^21^. However, the relationship among the Tenthredinoidea was stable no matter which matrix and method was used. We present the BI and ML results based on three matrices of the nucleotide sequences in Fig. 6.

Our analyses support the paraphyly and basal position of Symphyta in Hymenoptera and are congruent with traditional views, while Orussoidea forms a sister group with the suborder Apocrita^9^,^16^,^34^. There are three strongly supported lineages in the Symphyta, Tenthredinoidea, Cephoidea, and Orussoidea. Our analyses support a phylogenetic relationship of Tenthredinoidea + (Cephoidea + (Orussoidea + Apocrita)) in the Symphyta, as in most other analyses^9^,^20^,^21^. The analyses support the monophyly of Tenthredinoidea. Within the Tenthredinoidea, Tenthredinidae forms a sister relationship with Cimibicidae, while the family Pergidae forms a sister lineage to Cimibicidae + Tenthredinidae. Within the Tenthredinidae, the subfamilies Tenthredininae and Allantinae form a sister lineage and are then a sister to the Nematinae family, as revealed in Malm and Nyman^9^, and this is also well defined by morphology^20^,^21^.

## Materials and Methods

### Sample preparation and DNA extraction

A specimen of *T. anthracinum* was collected from Hanmi in Tibet, China, while a specimen of *A. rufocephalus* was collected from Songshan Forest Park in Yanqing County, Beijing, China. All the specimens were stored at −80 °C in 100% ethanol prior to DNA extraction. Total genomic DNA was extracted from legs of the specimens using the DNeasy tissue kit (Qiagen Hilden, Germany), following the manufacturer’s protocols. The voucher specimens are kept in the Evolutionary Biology Laboratory of Zhejiang University, China.

### Primer design, PCR amplification and sequencing

First, we used a set of universal primers for the insect mitochondrial genome^2^,^8^ for amplification and sequencing of partial gene segments. Then we designed specific primers based on the sequenced segments to amplify regions to bridge the gaps between different segments. PCR was carried out using Takara LA *Taq* (Takara Biomedical, Japan) with the following conditions: initial denaturation at 96 °C for 3 min and then 40 cycles at 95 °C for 30 s, annealing at 45–53 °C for 30 s, extension at 60 °C for 1–2 min, and final extension at 60 °C for 10 min. PCR components were added following the Takara LA *Taq* protocols. The PCR products were directly sequenced by TSINGKE Company (Beijing, China) using a primer-walking strategy from both strands.

### Mitochondrial genome annotation

The tRNA genes were initially identified using the tRNAscan-SE search server, with a Cove cutoff score of 5, and Mitos WebServer. The tRNAscan-SE search server set the parameters that the source was Mito/Chloroplast and the genetic code was the Invertebrate Mito genetic code, while Mitos WebServer set the parameters that the genetic code was the Invertebrate Mito genetic code. The tRNAs that could not be found using these two approaches were confirmed by sequence alignment with their homologs in related species. Protein-coding genes were identified by Blast searches in GenBank, according to other published hymenopteran mitochondrial genomes. The rRNA genes and control region were identified by the boundary of the tRNA genes and by comparing them with other insect mitochondrial genomes. Secondary structures of tRNAs were predicted using the tRNAscan-SE search server (Lowe and Eddy, 1997) and Mitos WebServer^45^, and others were predicted manually if they could not be found using the tRNAscan-SE search server.

### Comparative analysis of the mitochondrial genomes from Symphyta

We compared the mitochondrial genomes of 10 species from the Symphyta, including the two newly sequenced ones in our study. Features of gene arrangement, base composition, codon usage and base substitution of PCGs were analyzed. Since there were several tRNA genes not obtained in some species, we analyzed the base composition using the PCGs only.

The base composition was calculated by Mega6^47^. The AT and GC asymmetries, called the AT-skew and GC-skew, were calculated according to the formulas suggested by Hassanin *et al.*^41^: AT-skew = (A − T%)/(A + T%) and GC-skew = (G − C%)/(G + C%). The intergenic spacers and overlapping regions between genes were counted manually. The relative synonymous codon usage (RSCU) of all protein-coding genes was calculated by CodonW (written by John Peden, University of Nottingham, UK). We used the software package DnaSP 4.0^48^ to calculate the number of synonymous substitutions per synonymous site (Ks) and the number of nonsynonymous substitutions per nonsynonymous site (Ka) for each Symphyta mitochondrial genome, using that of *Ascaloptynx appendiculatus* from Neuroptera as a reference sequence.

### Phylogenetic analysis

#### Taxa selection

To investigate the phylogenetic relationships within the Symphyta, we used all eight species of Symphyta for which the mitochondrial genomes had previously been published, and the two newly sequenced mitochondrial genomes in this study (Table 1). The 10 species can be divided into three superfamilies, Tenthredinoidea, Cephoidea and Orussoidea. There were six species belonging to Tenthredinoidea, three species belonging to Cephoidea, and one species belonging to Orussoidea. Four mitochondrial genomes from the Apocrita were used to construct the phylogenetic tree, because of the close relationship between Apocrita and Symphyta. The four insects represented four families, four superfamilies and both parts of Apocrita. *Vanhornia eucnemidarum* (Vanhorniidae)^49^ and *Diadegma semiclausum* (Ichneumonidae)^43^ represented Terebrantia, while *Apis mellifera* (Apidae)^50^ and *Vespa bicolor* (Vespidae)^51^ represented Aculeata (Table 1). *A. appendiculatus*^52^ from the order Neuroptera was set as an outgroup because of the close relationship between Hymenoptera and Neuroptera.

#### Sequence alignment, data partition and substitution model selection

We used MAFFT version 7.205 for the alignment of protein-coding and RNA genes, which implements consistency-based algorithms^53^. We used the G-INS-I and Q-INS-I algorithms in MAFFT^54^ for protein-coding and RNA alignment, respectively. The alignment of the nucleotide sequences was guided by the amino acid sequence alignment using the Perl script TranslatorX version 1.1^55^.

Data partitioning, and the ability to apply specific models to different partitions, is ideal for analyzing mitochondrial genomes^3^. We used PartitionFinder version 1.1.1^56^ to simultaneously confirm partition schemes and choose substitution models for the matrix. The search models for DNA and amino acid sequences were set to be “mrbayes” and “all protein”, respectively. The greedy algorithm was used, with branch lengths estimated, to search for the best-fit partitioning model. The branch lengths were set as linked.

#### Phylogenetic inference

We constructed the phylogenetic relationships among the Symphyta with the Bayesian inference method (BI) using Mrbayes v3.2.5^57^, and the Maximum Likelihood (ML) method using RAxML v8.0.0^58^, based on the nucleotide sequences of the 13 protein-coding genes, 20 tRNAs (except *trnI* and *trnQ,* as they were missing in more than half of the 14 species) and two rRNAs. In the BI, we used the GTR+I+G/GTR+G/HKY+I+G/HKY+G model for nucleotide sequences and the MtArt+I+G+F/MtArt+I+G model for amino acid sequences (Table 5). Four simultaneous Markov chains were run for 10 million generations, with tree sampling occurring every 1000 generations and a burn-in of 25% trees. In the ML analyses, we used the GTRGAMMA and PROTCATMTART models for nucleotide and amino acid partitions, respectively. For each analysis, 200 runs were conducted to find the highest-likelihood tree, followed by analysis of 1000 bootstrap replicates.

## Additional Information

**How to cite this article**: Song, S.-N. *et al.* Comparative and phylogenetic analysis of the mitochondrial genomes in basal hymenopterans. *Sci. Rep.* **6**, 20972; doi: 10.1038/srep20972 (2016).

## Figures and Tables

**Table 1:** The mitochondrial genomes used in this study.

	Species	Superfamily	Family	Accession number	References
Ingroup	*Allantus luctifer*	Tenthredinoidea	Tenthredinidae	KJ713152	Wei, *et al.*^36^
	*Asiemphytus rufocephalus*	Tenthredinoidea	Tenthredinidae	KR703582	This paper
	*Monocellicampa pruni*	Tenthredinoidea	Tenthredinidae	JX566509	Wei, *et al.*^59^
	*Tenthredo tienmushana*	Tenthredinoidea	Tenthredinidae	KR703581	Song, *et al.*^60^
	*Trichiosoma anthracinum*	Tenthredinoidea	Cimbicidae	KT921411	This paper
	*Perga condei*	Tenthredinoidea	Pergidae	AY787816	Castro and Dowton^33^
	*Cephus cinctus*	Cephoidea	Cephidae	FJ478173	Dowton, *et al.*^27^
	*Cephus pygmeus*	Cephoidea	Cephidae	KM377623	Korkmaz, *et al.*^35^
	*Cephus sareptanus*	Cephoidea	Cephidae	KM377624	Korkmaz, *et al.*^35^
	*Orussus occidentalis*	Orussoidea	Orussidae	FJ478174	Dowton, *et al.*^27^
	*Apis mellifera syriaca*	Apoidea	Apidae	KP163643	Haddad^61^
	*Vespa bicolor*	Vespoidea	Vespidae	KJ735511	Wei, *et al.*^51^
	*Diadegma semiclausum*	Ichneumonoidea	Ichneumonidae	EU871947	Wei, *et al.*^62^
	*Vanhornia eucnemidarum*	Proctotrupoidea	Vanhorniidae	DQ302100	Castro, *et al.*^49^
Outgroup	*Ascaloptynx appendiculatus*	Neuroptera (Order)	Ascalaphidae	FJ171324	Beckenbach and Stewart^52^

**Table 2:** Nucleotide compositions in regions of the mitochondrial genomes used in this study.

Species	Accession number	Whole		PCGs		*rrnl*		*rrns*	
		Length (bp)	AT (%)	Length (bp)	AT (%)	Length (bp)	AT (%)	Length (bp)	AT (%)
*Allantus luctifer*	KJ713152	15418	81.13	11212	79.90	1340	84.10	807	83.52
*Asiemphytus rufocephalus*	KR703582	14864	81.40	11257	80.30	1339	85.06	803	83.44
*Monocellicampa pruni*	JX566509	15169	77.22	11176	75.20	1356	82.67	797	81.18
*Tenthredo tienmushana*	KR703581	14942	80.14	11278	79.00	1355	83.17	797	83.69
*Trichiosoma anthracinum*	KT921411	15392	80.76	11185	79.30	1351	84.46	800	83.50
*Perga condei*	AY787816	13413	77.92	10148	76.50	1357	83.05	728	80.91
*Cephus cinctus*	FJ478173	19337	81.96	11291	79.10	1386	84.63	1011	80.51
*Cephus pygmeus*	KM377623	16145	79.82	11293	77.50	1367	84.56	1008	84.42
*Cephus sareptanus*	KM377624	15212	79.20	11285	77.30	1379	83.76	1014	85.21
*Orussus occidentalis*	FJ478174	15947	76.21	11174	74.20	1336	80.09	787	81.07
*Apis mellifera*	KP163643	15427	84.18	11029	83.20	1366	84.55	781	81.31
*Vespa bicolor*	KJ735511	16937	81.72	11230	79.30	1451	84.77	838	85.20
*Diadegma semiclausum*	EU871947	18728	87.41	11122	83.70	1392	88.00	768	88.54
*Vanhornia eucnemidarum*	DQ302100	16567	80.14	11068	78.20	1327	82.82	395	80.76

**Table 3:** The AT- and GC-skew of the mitochondrial genomes used in this study.

Species	PCGs					
	T(U)	C	A	G	AT-skew	GC-skew
*Allantus luctifer*	45.0	10.2	34.9	10.0	−0.126	−0.010
*Asiemphytus rufocephalus*	45.1	9.7	35.2	10.0	−0.123	0.015
*Monocellicampa pruni*	42.2	12.9	33.0	11.9	−0.122	−0.040
*Tenthredo tienmushana*	44.6	10.6	34.4	10.4	−0.129	−0.010
*Trichiosoma anthracinum*	44.1	10.4	35.2	10.3	−0.112	−0.005
*Perga condei*	43.2	11.6	33.3	12.0	−0.129	0.017
*Cephus cinctus*	44.0	10.7	35.1	10.2	−0.113	−0.024
*Cephus pygmeus*	43.2	11.1	34.3	11.3	−0.115	0.009
*Cephus sareptanus*	42.8	11.6	34.5	11.1	−0.107	−0.022
*Orussus occidentalis*	42.0	13.7	32.2	12.1	−0.132	−0.062
*Apis mellifera*	46.1	8.6	37.1	8.3	−0.108	−0.018
*Vespa bicolor*	44.3	11.1	35.0	9.6	−0.117	−0.072
*Diadegma semiclausum*	47.0	8.2	36.7	8.1	−0.123	−0.006
*Vanhornia eucnemidarum*	42.7	11.7	35.5	10.0	−0.092	−0.078

**Table 4:** The mismatched base pairs of tRNA of the mitochondrial genomes from *Trichiosoma anthracinum* and *Asiemphytus rufocephalus.*

Species	tRNA	Mismatched base pairs	Stem	No	Species	tRNA	Mismatched base pairs	Stem	No
*Trichiosoma anthracinum*	*trnA*	U-U	AA	1	*Asiemphytus rufocephalus*	*trnA*	U-U	AA	1
	*trnE*	A-C	TΨC	1		*trnL2*	U-U	AA	2
	*trnG*	U-U	AA	1		*trnN*	A-A	TΨC	1
	*trnL2*	U-U	AA	1		*trnW*	U-U	AC	1
	*trnR*	U-U	AA	1					

**Table 5:** The partitions of the mitochondrial genome sequences identified by PartitionFinder.

Partitions	Models	Genes
P1	GTR+I+G	*a6p1*, *c2p1*, *c3p1*, *cbp1*, *n3p1*, *trnR*
p2	GTR+I+G	*trnA*, *trnC*, *trnD*, *trnE*, *trnF*, *trnG*, *trnH*, *trnK*, *trnL1*, *trnL2*, *trnM*, *trnN*, *trnP*, *trnS1*, *trnS2*, *trnT trnV*, *trnW*, *trnY*
p3	GTR+I+G	*a8p1*, *a8p2*, *n2p1*, *n6p1*
p4	GTR+G	*a6p2*, *c1p2*, *c2p2*, *c3p2*, *cbp2*, *n3p2*
p5	GTR+I+G	*n1p2*, *n4lp2*, *n4p2*, *n5p2*
p6	HKY+G	*n2p3*, *n6p3*
p7	GTR+I+G	*n1p1*, *n4lp1*, *n4p1*, *n5p1*
p8	GTR+I+G	*n2p2*, *n6p2*
p9	HKY+G	*n1p3*, *n4lp3*, *n4p3*, *n5p3*
p10	HKY+I+G	*a6p3*, *a8p3*, *c1p3*, *c2p3*, *c3p3*, *cbp3*, *n3p3*
p11	GTR+G	*rrnl*, *rrns*
p12	GTR+I+G	*c1p1*
